# “NE@R”: a new resource to promote preterm infants’ development through parents-delivered guided play

**DOI:** 10.3389/fpubh.2025.1597244

**Published:** 2025-07-03

**Authors:** Sonia Trussardi, Cecilia Naboni, Camilla Caporali, Camilla Pisoni, Stefano Ghirardello, Simona Orcesi

**Affiliations:** ^1^Child Neurology and Psychiatry Unit, IRCCS Mondino Foundation, Pavia, Italy; ^2^Department of Brain and Behavioral Sciences, University of Pavia, Pavia, Italy; ^3^Neonatal and Intensive Care Unit, Fondazione IRCCS Policlinico San Matteo, Pavia, Italy

**Keywords:** early intervention, preterm birth, play, neonatal intensive care, parent experience, digital resource, environmental enrichment

## Abstract

**Introduction:**

The huge prevalence of neurodevelopmental disorders underscores the necessity for novel, comprehensive prevention strategies for neuroprotective intervention, particularly in preterm infants. The COVID-19 pandemic has accelerated the transformation of healthcare services, emphasizing the use of digital resources. Given the rapid brain development in infants in the first 1,000 days of life and the demonstrated impact of adaptive neuroplasticity, the implementation of early and ecological interventions are essential for supporting optimal neurodevelopment in this vulnerable population. Aim of this project is to develop a digital tool for parent-led parent-based intervention and assess its feasibility and accessibility.

**Materials and methods:**

We collected evidence on early intervention strategies for preterm infants through a non-systematic review of current literature to develop the platform and created an ad-hoc questionnaire to evaluate the tool’s feasibility and acceptability in our neurological follow-up.

**Results:**

“NE@R” is a digital platform designed to support neurodevelopment through parents-delivered play. The platform offers evidence-based information, videos, and practical activities to enhance motor, cognitive, social, and language development at each developmental phase. We introduce the resource in our clinical setting and collect 100 preterm infants’ families feedback. The majority of parents reported finding the resource beneficial, with many expressing increased confidence in supporting their child’s development.

**Discussion:**

Preterm babies families’ support represents a precious field of intervention both for parents and infants at risk. “NE@R” has proven to be an effective, low-cost tool within our follow-up program, aligning with the principles of family-centered care.

## Introduction

Prematurity is a condition that impacts approximately 10% of newborns every year worldwide (1). This huge population needs to be monitored in the field of early detection and early intervention of neurodevelopmental disorders: the preterm birth exposes the infant to a higher susceptibility risk for brain damage and maturation. Besides the effect of the primary brain damage, advances in neuroimaging have proved the existence of secondary dysmaturative effects occurring also in preterm babies without a major brain impairment. Dysmaturation can occur both in association with white matter injury, causing hypomyelination or impairment of gray-matter structures (“encephalopathy of prematurity”) and as a primary effect, for example with a reduction of the dendritic development of cortical gray matter (2). Moreover a preemie’s brain is exposed too early to the environment, with a negative impact on usual nurturing experiences shaping developmental trajectory. This can be caused both by direct negative experiences, like pain and excessive sensory stimulus, and by the lack of protective experience provided by the continuous multisensory and emotional contact with parents (3). In the past decades, the growing field of neuroscience has shown the powerful effects of environmental enrichment in modeling the developing brain. Neuroplasticity can be defined as the dynamic biological capacity of the central nervous system to undergo maturation, change its structure and function in response to experiences and to adapt following injury. This incredible capacity is achieved by modulating subsets of genetic, molecular and cellular mechanisms: they produce continuous changes in synaptic connections and neural circuitry formation (4). The context of ongoing neural development and the increased vulnerability to adverse outcomes due to immaturity itself, make it impossible to predict a baby’s outcome after a brain damage. Indeed, in childhood neurology it is impossible to make exact previsions based only on the story of birth or on neuroimaging: the individual developmental trajectory depends on how neuroplasticity has worked to repair and support brain maturation (5). Following the concept of “early as possible,” there is growing interest in developing neuroprotective care programs, that target from birth and during NICU stay environmental factors that can modulate baby’s cerebral maturation: protection of sleep, reduction of pain and stress, promotion of visual and auditory modulated experiences, postural care and enhancement of parents responsive parenting (6). About last post-discharge early intervention evidences, interventions that target parent-infant relationships result as more effective the ones that focused only on the baby or only on parents. Moreover, early intervention on preterm babies can provide improvements in motor and cognitive outcomes, especially the ones that start in the NICU and continue at home and involve parents actively (7). In fact, the primary environment of a baby is family: an infant’s growth process cannot be fully understood without including his parents and social context, which is crucial for neurodevelopment (8). The style of daily interaction between infants and their parents can have an objective impact on babies brain: for example, a recent study found sensitive parenting correlates with major subcortical brain development (9), while intrusive parenting is associated with smaller cortical brain volumes and altered white matter microstructure (10). In case of emergence of a specific neurodevelopmental disorder, specific rehabilitative intervention must be started as early as possible and targeting the integration of all areas of development. For example, a systematic review about main effective interventions for children with cerebral palsy point as necessary features some concepts: early, intense, enriched, task-specific, training-based intervention, with increased improvements when the intervention is personalized to a baby’s home enjoyment (7).

The Italian Neonatal Society (SIN) has recently revised the national follow-up standards. In this guidelines, authors indicate to monitor the neurodevelopment of all preterm babies born <32 weeks of g.e. and <1,500 gr or with other risky conditions from birth to 6 years. First screening evaluations include neurological evaluation, neurobehavioral observation and qualitative observation of developmental profile. The aim is to detect early signs of risk and to monitor their evolution, in order to allow appropriate early interventions. Moreover, there is evidence to support all preterm babies with an individualized habilitative program, that is able to guide positive and age-targeted experiences and a social and physical environment. This intervention can have a protective effect on fragile brains and “preterm parents.” It is fundamental to create a specialized multidisciplinary equipe that is able to observe and integrate information of every single baby to a major comprehension of his history (11). In Italy, the “Neuro and Psychomotor Therapist of Developmental Age” (TNPEE) is a clinician specialized in the prevention, assessment and rehabilitation of children with neurodevelopmental disorders, working in partnership with other professionals throughout the child’s development. The above professional profile implies an in-depth knowledge of neurodevelopment and ability to integrate and focus in the interplay between all areas of development, including motor, cognitive, social, emotional and communication, in a major comprehension of babies resources, needs and desidere at each developmental age. This specialized therapist uses play as a tool to involve babies, to create action and interaction, to promote changes in the area of proximity of a child (12).

During the last years, some changes occur in usual preterm care and follow-up due to the dramatic covid-19 pandemic: restrictions imposed damage the frequency of visits and reduce the possibility to meet families in person. In addition, parent-infant daily play routine changed: for example, parents were obliged to spend more time at home and less outside with their babies, hygienic precautions force the decrease of usual contact and oral and tactile free exploration, isolation caused the absence of early interactions with peers and meeting with strangers. Moreover, the perception of a stressful situation and the psychological effects of social isolation and sense of loneliness caused many psychological effects on people, with possible effects on parental self-efficacy (13) and consequently on parents’ quality in caring for their infants (14). Because of these factors, in clinical services there was a rapid increment of the implementation of new digital care strategies to maintain and improve guidelines applications. A recent review about the use of mobile technology for preterm babies states that this approach can be useful for parents’ education and support (15), even once the pandemic emergency has ended. There are few existing mobile apps targeting the transition from NICU to home and even fewer apps that have been produced with the direct contribution from clinicians and studied in a clinical setting (16). Moreover, many tools are in the English language and focus mainly on daily care procedures or only on motor exercises (17), lacking the play-interaction dimension that supports development in all fields. To our knowledge, there are no available platforms in our country that have been developed specifically for preterm infants to support neurodevelopment through parent-infant play and to implement habilitative intervention follow-up guidelines in clinical practice.

### Aim

For these purposes, the first aim of this study was to collect best practice evidence about neurodevelopmental support in preterm infants and integrate them with our group clinical experience. Secondly, we develop a new digital tool where to include all practical information to promote specific parent-implemented play-based intervention. Finally, we decided to assess the tool feasibility and acceptability in our clinical setting during the covid-pandemic period and present its usefulness even after this historical period for implementing standard care practice in Follow-Up Program for the Newborn at neurological risk at IRCCS Policlinico S. Matteo Hospital in Pavia, Italy.

## Materials and methods

### Platform and questionnaire design

The project was conceived with the aim of developing a unique tool tailored for preterm infants, while maintaining a developmental support focus that includes general guidelines for promoting development. Despite this, it is designed to become an integral component of the post-discharge care program for families of infants “at high risk.” Therefore, it necessitates an individualized assessment of the intervention’s feasibility, based on the specific needs and characteristics of each family unit. For this reason, we decided not to use a publicly accessible website, but to choose a private platform that can directly be shared by the professional to the family during the visit.

A brief review of the main digital platforms currently available was conducted searching on the web: positive and negative aspects were compared to find the best platform for our purpose between many available, like Linoit, Netboard, Wakelet, Stormboard, Edmodo and Padlet. The comparison was based on some characteristic that were identified as basical by clinicians: quick and anonymous access, availability of a free app to provide access on smartphones, an Italian version to facilitate comprehension, support for various types of files to include multimedia content, allow progressive expansion of the platform’s content over time with an in time update and easily programmable with personalizable graphics and layout to make it credible and motivating for parents. The selected platform allows for good customization of the internal structure and thus the way the content is presented. The ability to customize colors, size and type of content (written form, PDF, video files, audio files) allows an adaptation of the resource to the needs but also to the characteristics of the population for which it is conceived and designed. With respect to user experience, it is a platform accessible via browser or specific app (Padlet) and navigable from any technological device (computer, tablet, cell phone) with ease. Content can be viewed on the platform and, in some cases selected by staff, can be downloaded in PDF format. The platform-selection process was made directly by the neurodevelopmental therapists that created the contents, in order to identify what digital support can better fit with practical purposes since the beginning. We chose “Padlet” to be the best support for our clinical aim, as it was the only one that allowed all these features.

As regarding the contents, we integrated information coming from different sources of literature about preterm preventive and habilitative interventions (18–20) with informative materials already used in our clinical setting, such as the contents written in the clinical book “Small steps to grow together.” This book has been written in past years by our clinical team as a support for families and contains some information about transition from NICU to home and general advice about how to support neurodevelopment from 0 to 12 months. Moreover, we revised the national follow-up standards (11) and verified coherence between our contents and suggested habilitative intervention strategies. Finally, we collect practical play activities that are mainly shared during typical follow-up visits by our team and try to structure them according to age and main purposes, integrating previous written materials with our developed experience in parents-infant play and early intervention. The platform design derives from clinical group discussion about how to implement our contents in a feasible and independently accessible tool for parents. We decided to insert both general information about our clinical follow-up and prematurity and structure progressively deeper structures about specific themes: all contents are organized in a progressive sequence, from general statement to practical play-activity that supports that area of development. For these purposes, we decided to both include written information and multimedia materials, like images or video, particularly for those that are more easily shared with visual representation. To assess the reliability and feasibility of the tool in supporting parental competencies and promoting psychomotor development in at-risk newborns, we planned to develop an “*ad hoc*” satisfaction questionnaire as an outcome measure to qualitatively investigate caregivers’ perceptions regarding the usability and usefulness of the platform. The questionnaire was developed for this project and designed to be administered directly through the platform, providing direct access to a Google Form. In this way, the results are automatically and anonymously submitted to a system exclusively monitored by professionals. This first version of the questionnaire does not include child’s clinical data or parents’ background information; we only derived infants’ age period at the moment of access to the platform selected by the parents. We identify 3 main areas of interests: platform accessibility, play and advice utility perceived and parent–child play-interaction empowerment. To cover the complexity of these purposes, we decided to insert 2 different types of questions: Multiple-choice questions and short-answer open questions.

### Statistical analysis

We use the R studio (R 4.4.0) and Jasp 0.19.3.0 program to analyze numerical and categorical variables in our dataset. Categorical variables are analyzed using frequencies and percentages. The Pearson correlation coefficient is used to explore possible correlation between the collected variables. Qualitative values are converted into quantitative ones with coherence with positive and negative significance.

## Results

### NE@R: newborn dEvelopmental @t home resource and questionnaire

The newly created platform is called “Newborn dEvelopmental @t home Resource (NE@R)” with reference to digital innovation and the desire to enhance proximity with parents of our clinic. We developed a multicomponent tool: 6 composed platforms, each regarding subsequent periods of development from birth to 24 months corrected age and strictly connected with usual timeline follow-up visits (0–3 months, 3–6 months, 6–9 months, 9–12 months, 12–18 months, 18–24 months). The platform offers evidence-based information, videos, and practical activities to enhance motor, cognitive, social, and language development at each developmental phase. Each platform contains common information about basic knowledge regarding preterm development, for example the concept of corrected age and the importance of play. Moreover, contents are organized in 4 main areas:

- Postural and Motor Organization: Focused on promoting motor initiative, discouraging the development of asymmetries and abnormal postures, and minimizing the influence of transient signs.
- Exploration and manipulation: Aimed at supporting diverse and shared play experiences in the home, encouraging oral and manual exploratory strategies, enhancing hand-eye coordination and fine motor skills, and fostering the development of flexible and adaptive cognitive strategies.
- Relational and Communication Aspects: Designed to strengthen the parent–child relationship, guide early self-regulation strategies, and enrich facial, gestural, and verbal expression.
- Developmental Support Tools: Providing parents with guidance on optimal environmental structuring to support emerging skills at each stage of the child’s development.

**Figure 1:**
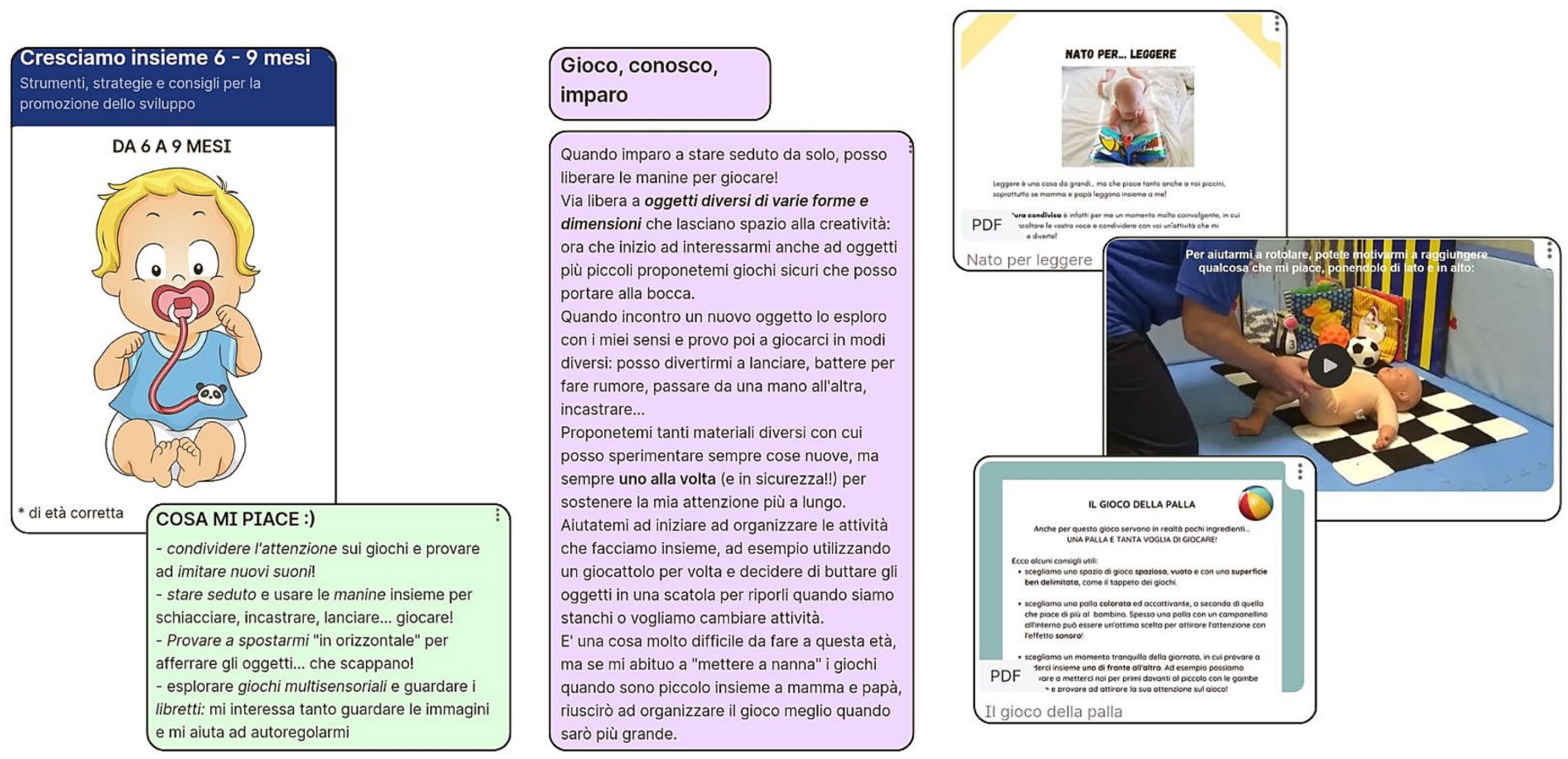
Content representation. Representative graphics of the content included in the platform, with reference to the developmentally correct 6–9 months age group. From left: overview of the developmental period and content highlights for parents (e.g., “what I like”: the child speaks in the first person and tells the main strategies to support neurodevelopment); an example of descriptive content in which the child tells about his evolving development in that specific area (postural and motor skills); examples of content presented in different formats: simple explanation of the importance of reading to support promote language; explanatory video of how to encourage rolling in this period of life; example of a “tutorial” game to encourage exchange play between parents and children.

In each section, parents can have access to evidence-based information about how to understand milestones complexity and role, practical activities to be easily reproduced at home with common objects to support them. Contents are provided both on a written form or on video recorded by therapists (Figure 1).

The “*ad hoc*” satisfaction questionnaire (Supplementary Data Sheet 1) consists of 21 questions (multiple-choice or short-answer) that explore parents’ opinions on the access methods and digital format, the informational quality of the content and the perceived support provided by the platform both on infant and parents themselves. Finally, the questionnaire collects any recommendations or suggestions for improving the tool and its potential use as an integration to the standard care practices in clinical settings in the future.

### Application in our clinical setting

**Figure 2:**
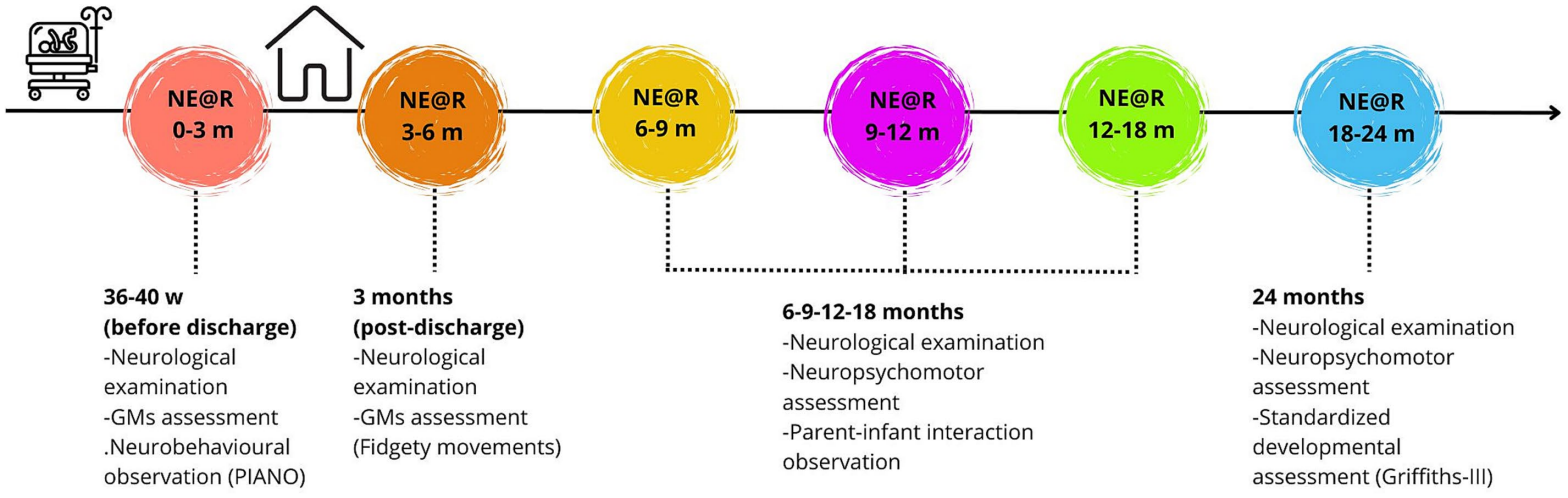
Follow-up timeline and integration of NE@R project.

We started by sharing NE@R in our follow-up program to families with babies born preterm (<37 weeks of gestational age). About 230 Italian speaking families who attend neurological visits between 1 November 2020 and 31 December 2023 (i.e., both during the pandemic period and in subsequent months) received the access to the age-related platform by a QR Code. The 0–3 month module was introduced to parents in the Neonatal Intensive Care Unit (NICU) during the final pre-discharge visit. A TNPEE presented the project, guiding parents through the platform’s features on a smartphone to address any immediate questions and ensure effective independent use. After discharge, subsequent platform modules were provided at each developmental follow-up visit, with QR codes aligned to the child’s current developmental stage (Figure 2). Main useful activities and resources for the specific child were shared to parents on the platform based on emerging abilities. The platform’s content is specifically tailored for families under clinical care: parents were encouraged to share it with extended family members and caregivers and then provide us with feedback by completing the online questionnaire on anonymous form.

Regarding the satisfaction questionnaires, we received them filled out by 100 families, mainly by mothers (93%). There is a different distribution of answers referring to age platforms 12% of 0–3 months, 29% of 3–6 months, 25% of 6–9 months, 9% of 9–12 months, 17% of 12–18 months, 5% of 18–24 months.

In the first section, related to the technological access to the platform, the majority of parents declare to prefer the digital format (72%) and access the platform mainly by smartphones (89%); the 70% of parents think that the platform is easy to use and only 10% reporte some difficulties. Families enter the platform mainly from 1 to 5 times (52%) in the period that occurs after the platform presentation and the following Follow-Up visit assessment.

In the second section, related to the perceived usefulness of the resource, the majority of parents declare to know only some of the contents before (54%) and try to try to reproduce the majority (67%) of the plays described in the platform. Moreover, the 63% report having found additional play ideas to support specific areas of frailties discussed during the developmental assessment.

In the third section, regarding parents and infants play involvement, the majority of parents think that the resource is able to provide new ideas to play together with their baby at home (“a lot” or “very much” 73%). They report higher parents (““a lot” or “very much” 87%) and children (“al to” or “very much” 79%) enjoyment. Moreover, the totality of parents think that the platform is useful in addition to standard follow-up visits (“A lot” or “very much” 100%).

A special section collects suggestions to improve the platform: 43% of families ask for more practical play activities and 33% for additional videos and images. Many families ask for the increase of information regarding general babies development (28%) and specifically preterm development (30%). 23% of parents suggest implementing advice about age-related toys. The 18% of parents suggest improving graphical design and the 18% the access modality; the 2% of parents suggest the reduction of written information.

**Figure 3:**
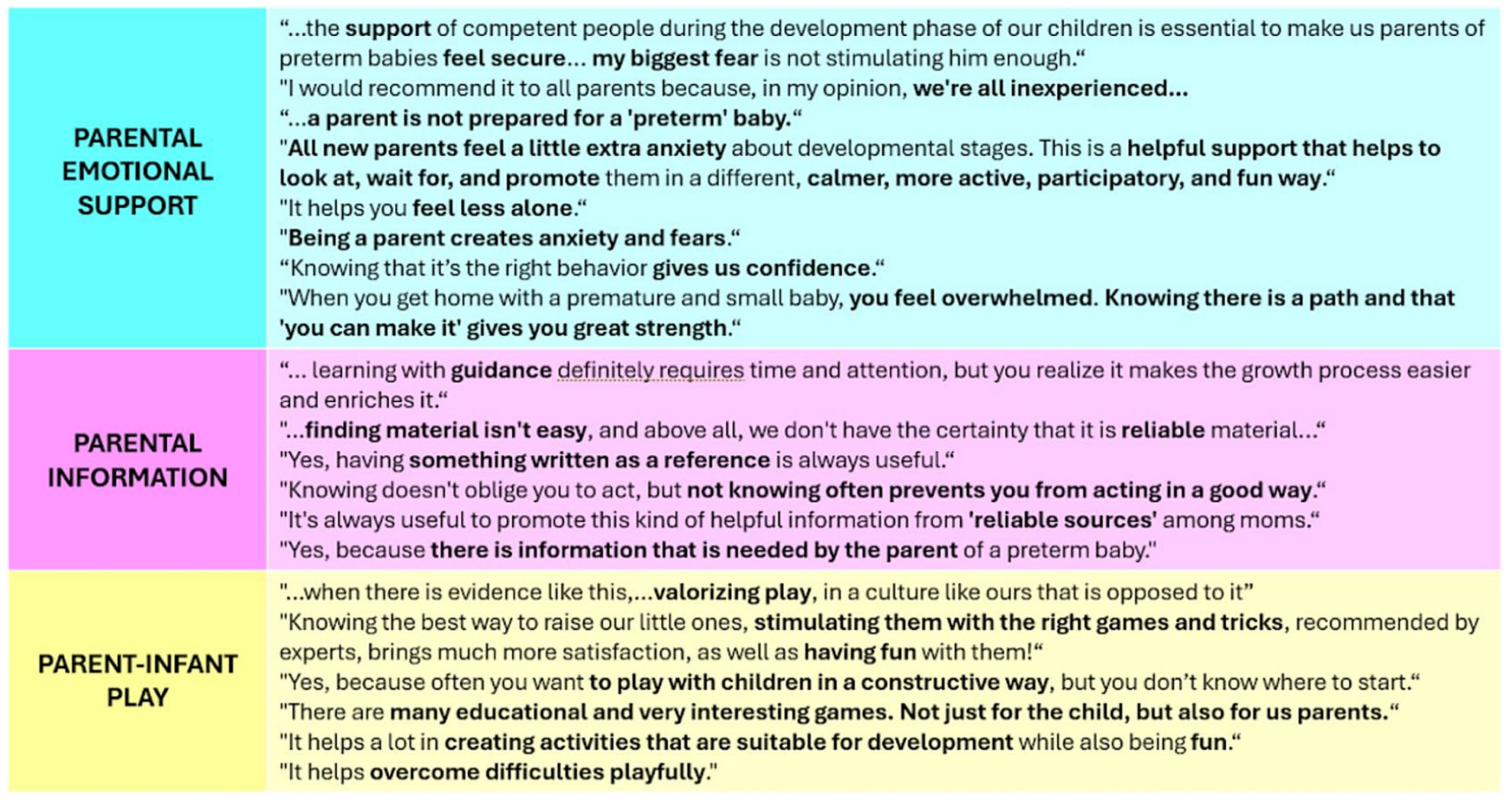
Parents’ main significant opinions organized into 3 main recurrent themes: Parental emotional support, Parental information and Parent-infant play.

Open questions were useful for clinicians to better understand practical examples provided by families about their perspective on the project and needs. Three main themes emerged from the thematic analysis of parents’ answers (Figure 3).

Pearson correlation performed shows a positive correlation between parent’s enjoyment during play (“Did you enjoy playing with your child?“) and perceived child enjoyment (“Do you think the activities suggested were enjoyable for your child?”) (*p*-value < 0.01). Both this variables were also statistically correlated with the number of times the platform was consulted (“How many times did you consult it between one visit and the next?“) (*p*-value < 0.01) the perceived help in promoting play at home (“Do you think this tool has provided you with new ideas for playing together at home?”) (*p*-value < 0.01) and the perceived usefulness of the platform in addition to standard visits (Do you think this platform is useful in addition to the traditional follow-up meetings?) (*p*-value < 0.01).

The perceived platform accessibility and usability (“Did you find the tool easy to consult?”) was strongly correlated with the perceived usefulness of the platform (*p*-value < 0.015) and the number of playing activities tried at home (“Did you try any of the activities and games suggested“) (*p*-value < 0,030). The number of playing ideas is also correlated with the perceived child enjoyment (*p*-value < 0.01).

Families who know less contents before using the platform (“Were you already familiar with the advice provided for this age range?”) perceived the tool as more useful (“Do you think this platform is useful in addition to the traditional follow-up meetings”) (*p*-value: 0.008) and as a valid support for new ideas for at home play (*p*-value < 0.01) A positive correlation is found also between the possibility to find specific activities to support frailties discussed during the visit, the number of activities done at home (*p*-value 0.002) and the perceived usefulness of the platform in addition to standard visit (*p*-value < 0.01).

## Discussion

Modern cognitive science approaches consider an individual as the result of a complex interaction between genetics, epigenetics, neurobiology and environmental experiences; all these factors are interconnected in a complex system that changes over everyone’s life. According to general system theory, systems are the whole that emerges from the interaction of their parts (21): we cannot consider a single aspect without losing the complexity of the system. This concept opens the doors to a more feasible and efficient model to understand babies’ typical and atypical development, especially considering the extremely fast and complex dynamics caused by neuroplasticity in the first months of life.

The high accessibility of mobile devices for families in our society supports the possibility of using them not only as a source of connection, but also as a resource for finding useful knowledge. The COVID-19 pandemic increased this process and also induced clinicians to find alternative ways to communicate with patients: the use of mobile devices is gradually becoming a feasible device to connect with a wide population. Many parents of premature babies research information during and after NICU stay about how to support babies development on the internet (22). Nevertheless, they do not always find evidence-based information tailored for their baby specific needs and phase of development. This can lead to the loss of a valuable opportunity to act directly on adaptive neuroplasticity and positively influence a baby’s developmental trajectory.

Clinicians must actively provide early intervention programs that support every preterm baby’s development, due to their increased risk for neurodevelopmental disorders. Neuroscientific discoveries support play-based intervention that provide effective synaptic plastic changes thanks to child engagement: shared-emotions and motivation are at the base of effective neural learning. Play is the child’s dimension where it is possible to explore the environment, learn to act, develop self-determination and interact with others in a safe and engaging context (23). For a TNPEE, play is the main tool to observe a baby’s skills and motivations, evaluate possible changes through a relational mediation and actively shape babies growth through specific task-oriented sessions of play. Play is also a unique chance for parents to engage with their children and reinforce their relationships. By observing or joining their children in play, parents gain a rare insight into their child’s view of the world and needs. This concept is especially true for little babies, who cannot yet express themselves by verbal language and behavior is their main source of communication and interaction. This perspective allows parents to improve their communication and offers another space to provide gentle, caring guidance and to enhance connection (23). Families of preterm babies often express the desire to be supported in their pathway as parents: open answers from our sample highlight the presence of many themes connected with the emotional sphere, like the experience of anxiety, fear, perception of inexperience and loneliness. Nevertheless, parents also spontaneously report positive emotions connected with the usage of this digital tool, such as the perception of the presence of a guidance, the increased consciousness about knowledge and the power to make more informed choices. Besides primary aims, this project has been useful to share a “culture of play” and to provide parents practical instruments to understand the importance of parent-infant play and experience that in their home. Shared play has an effect both on parents and children: in our study families and children perceived enjoyment, supporting the idea of the presence of positive emotional connection during play experience. Moreover, mutual enjoyment has been correlated with an increased use of the platform. Our preliminary experience with NE@R confirmed the feasibility of our proposal and allowed us to highlight certain aspects. Even if we do not have demographic information about these families or children’s clinical data collected, our findings reveal an important difference between mothers and fathers’ involvement: the resource is directly presented to both parents but many times women are the ones who have to follow baby pathways and play. This suggests the necessity to create specific programs to increase fathers’ involvement and participation, in particular for preterm infants. Moreover, parents who find it easier to use the platform are also the ones who tried more playing ideas and consequently appreciate more their child’s enjoyment: this suggests the necessity to revise and continuously improve the tool design and graphics to increase parents participation. Families who perceive the platform as useful in addition to standard care also declare having found playing activities to support specific areas of development discussed during the visit: this strengthens the importance of providing practical information to guide parents, but always according to clinical evaluation of specific needs. Parents who declare to benefit most from this project are the ones who do not have much previous knowledge about preterm babies development: reliable information seems crucial for families to feel supported and capable of providing their children all the positive opportunities for their development.

### Limitations

Some limitations of the study regard the fact that the questionnaire is voluntarily and anonymously filled: there is a possible bias about not receiving feedback by families who are not so involved in the project. Other possible limitations regard the language barrier: cultural contents need to be translated and adapted for non-Italian speaking families in future versions. This first study was designed to study feasibility and applicability of NE@R in our clinical context and parents perspective; future studies will include a deeper collection of data about the sample in order to investigate possible correlation between children features (gestational age, weight, cerebral ecography, etc.) and the resource use and possible positive effects on development. As a matter of fact, our preliminary study does not include comparison groups between families who use the platform or do not join the project or, a detailed champion of preterm babies included. Moreover, in the future it will be interesting to assess this intervention effect with specific measures of outcomes about parent-infant relationship and infant development. Lastly, we performed simple statistical analysis of the data collected: it would be beneficial in further studies to collect wider data in order to perform stronger analysis such as multivariate and regression analysis.

## Conclusion

NE@R has become an effective, low-cost tool in our follow-up program, aligning consistently with the family-centered care approach and with new follow-up standard care goals. Future studies will investigate the possible impact on infant development and on parental self-efficacy and the effect of new updated versions of the platform, following parents suggestions and needs expressed.

## Data Availability

The raw data supporting the conclusions of this article will be made available by the authors, without undue reservation.
