# Supplementary material for: “NE@R”: a new resource to promote preterm infants’ development through parents-delivered guided play
Source: Front Public Health. 2025 Jul 3;13:1597244. doi: 10.3389/fpubh.2025.1597244 (PMC12267281; doi:10.3389/fpubh.2025.1597244)
Supplement: Supplementary file 1 [file Data_Sheet_1.PDF]

QUESTIONNAIRE

General informations

1. **I am completing this as...**
   - o Mother
   - o Father
   - o Grandmother/Grandfather
   - o Legal guardian
   - o Other
2. **Child's chronological age** ………
3. **Child's corrected age** ……….
4. **Age range consulted on the Padlet**
   - o 0-3 months
   - o 3-6 months
   - o 6-9 months
   - o 9-12 months
   - o 12-18 months
   - o 18-24 months

I SECTION: platform access

5. **What device did you use to consult the Padlet?**
   - o Mobile phone
   - o PC
   - o Tablet
6. **Do you believe that the digital format made using this tool more convenient and faster?**
   - o Not at all, I would have preferred a paper booklet
   - o Somewhat, but I would have preferred to also receive a paper booklet
   - o Yes, very much, I prefer the digital format
7. **Were you able to easily access the platform?**
   - o Not at all, I could not access it
   - o Somewhat, I encountered some difficulties
   - o Yes, the access was simple and quick
8. **How many times did you consult it between one visit and the next?**
   - o Never
   - o Once, as soon as I was given it
   - o Between 1 and 5 times
   - o Between 5 and 10 times
   - o More than 10 times
9. **Did you find the tool easy to consult?**
   - o No, I could not figure out how to find the information
   - o Somewhat
   - o Yes, the layout is clear and intuitive

II SECTION: Play and advices utility perceived

10. **Were you already familiar with the advice provided for this age range?**
    - o No, none
    - o A few
    - o Some
    - o Most
    - o All
11. **In particular, is there any information you had never been told about before, which you found useful?** ………………..
12. **Did you try any of the activities and games suggested?**
    - o No, none
    - o A few
    - o Some
    - o Most
    - o All
13. **During the previous follow-up meeting, were you given any specific advice to promote a particular skill in your child?** ……………………………………….

14. **If you answered yes to the previous question, did you find any helpful advice on the platform to support it?**

- No, I did not find any useful advice
- Somewhat, I found the advice I had already received during the in-person meeting
- Yes, I found additional helpful game ideas to promote it

15. **If yes, have you observed any improvements in this skill thanks to the games you did together? Can you provide an example?** ……………………

<u>III SECTION: Parent-child play-interaction empowerment</u>

16. **Do you think the activities suggested were enjoyable for your child?**

- No, not at all
- A little
- Somewhat
- A lot
- Very much

17. **Do you think this tool has provided you with new ideas for playing together at home?**

- No, not at all
- A little
- Somewhat
- A lot
- Very much

18. **Did you enjoy doing these activities with your child?**

- No, not at all
- A little
- Somewhat
- A lot
- Very much

19. **Do you think this platform is useful in addition to the traditional follow-up meetings?**

- No, not at all
- A little
- Somewhat
- A lot
- Very much

20. **Would you recommend this tool to another parent of a "preterm" child? Please justify your answer** …………

21. **To improve the platform, would you suggest we...**

- Modify the access methods to the platform
- Change the layout
- Reduce the amount of written information
- Add more images and videos
- Add more general information on your child's development
- Add more information on preterm development
- Provide more specific advice on age-appropriate games
- Add more suggestions for play activities
